# Kind Extinction: A Procedural Variation on Traditional Extinction

**DOI:** 10.1007/s40617-023-00833-w

**Published:** 2023-07-06

**Authors:** Courtney Tarbox, Jonathan Tarbox, Taira Lanagan Bermudez, Erin Silverman, Lauren Servellon

**Affiliations:** 1 FirstSteps for Kids, Los Angeles, CA USA; 2 University of Southern California, Los Angeles, CA USA

**Keywords:** extinction, kindness, compassion, applied behavior analysis

## Abstract

Operant extinction has substantial evidence to support its effectiveness across a variety of populations and behaviors. However, extinction procedures may be less-preferred by learners, caregivers, other community stakeholders, and the staff implementing them. In the current study, we evaluated the effectiveness of a “kind extinction” procedural modification, in which we provided a functionally arbitrary reinforcer in the form of genuine positive regard and validation, contingent on interfering behavior, while implementing escape and tangible extinction. The procedure produced large and rapid decreases in interfering behavior, accompanying increases in alternative behavior, and was rated as acceptable by caregivers and staff. Implications for increasing the social validity of behavioral procedures, as well as contributing to a more kind and compassionate future for the field of applied behavior analysis are discussed.

A substantial body of research has supported the effectiveness of operant extinction (Lerman & Iwata, 1996) and extinction is considered one of many critical components of comprehensive evidence-based behavioral intervention for autistic individuals (Huete et al., 2014). Despite the overwhelming scientific evidence in favor of the effectiveness of extinction procedures, extinction is not without limitations. Extinction can be difficult to implement, especially for escape-maintained behavior. Early procedural versions of escape extinction prescribed immediate physical guidance contingent on interfering behavior (Iwata et al., 1990). Physical guidance can be challenging to implement with a learner that is larger and/or stronger than the implementer but, more important, physically limiting a learner’s movement for therapeutic purposes is disconcerting from an ethical standpoint. Behavior analysts have an ethical responsibility to use the least restrictive procedures that are likely to be effective (Behavior Analyst Certification Board, 2020, Code 2.15), of minimizing or eliminating physical guidance (Association for Behavior Analysis International, 2010), and of treating others with compassion (Behavior Analyst Certification Board, 2020, Core Principle 2).

Taylor et al. (2019) defined compassion as action that is aimed at ameliorating another person’s suffering or distress. All applied behavior analytic procedures might usefully be reevaluated in terms of whether they evoke distress and it seems possible that extinction may be a productive place to start. Extinction can cause extinction bursts, which can include a temporary escalation of the behavior, as well as the occurrence of negative emotional responding (Fisher et al., 2022). Behavioral escalation and negative emotional responding could reasonably be interpreted as distress, so extinction procedures may be one context in which behavior analysts have an opportunity to take action to ameliorate that distress in the human beings who we are entrusted to support.

In addition to extinction potentially increasing learner distress in some cases, extinction may have low social validity (Pemberton & Borrego, 2007). That is, extinction procedures may have low social acceptability to learners, their caregivers, and other community stakeholders, and interventions not incorporating extinction may be more preferred by those actually experiencing the interventions (Owen et al., 2021). From a purely functional, process-based standpoint, there is nothing cold or unkind about a basic principle of learning; extinction is a basic learning process that applies to all operant behavior. But the specific *ways in which extinction procedures are implemented* can send negative messages to community stakeholders and to learners themselves. Although most ABA practitioners could tell you that extinction for behavior maintained by tangible reinforcers consists of withholding the tangible reinforcer contingent on the behavior, many practitioners also withhold attention or other forms of interaction when implementing extinction. For example, if a child engages in a tantrum that is motivated by access to a toy, ABA practitioners may implement extinction by withholding tangible reinforcement (i.e., access to the toy) during the tantrum while also refraining from providing attention to the behavior, effectively ignoring the child. Journal articles that describe extinction or differential reinforcement procedures commonly describe procedures that, by default, involve not interacting with the learner, other than to reinforce specific target alternative behaviors (Petscher et al., 2009). That is, target interfering behavior results in *no interaction* with the learner, target replacement behavior results in access to the functional reinforcer, and no other interaction between the therapist and learner is to occur, other than preventing physical safety concerns.

Social validity is a core value of the field of ABA (Wolf, 1978). According to Wolf, “By giving the same status to social validity that we now give to objective measurement and its reliability we will bring society into our science, soften our image, and make more sure our pursuit of social relevance” (p. 207). Wolf also emphasized considering whether the ends justify the means. For example, do the participants, caregivers and other consumers consider the treatment procedures acceptable? Wolf proposed this call to action 45 years ago and some have wondered if we, as a field, have adequately heeded the call (Callahan et al., 2017). If we have not adequately addressed the social validity of our extinction procedures, it may have practical consequences. When we ask caregivers to implement extinction with their children, we are expecting a great deal from them. Often, we are asking caregivers to commit to withholding a reinforcer that they have a long history of providing to their child. In a real sense, the behavior–reinforcer relation in that child–caregiver interaction may be an important part of their relationship, even if it may be viewed as maladaptive by behavior analysts who are trying to help the child learn alternative behaviors. In other words, from the caregivers’ perspective, reinforcing their child’s behavior may be perceived as a way of caring for or showing love to their child. In essence, when we ask caregivers to implement extinction, we are asking them to do something terribly difficult now, in hopes that it will lead to a better outcome later. Of course, extinction may take time, and even a short period can be difficult for caregivers to adhere to. We are asking caregivers to forgo the short-term negative reinforcement they get by discontinuing their child’s discomfort, in order to access the long-term positive reinforcement of their child having less interfering behavior and therefore a better life in the future. Although it is entirely possible that this choice could be “worth it” to caregivers, it raises the question: What if behavior analysts could be equally effective in decreasing interfering behavior, while making the procedures less aversive to implement?

One treatment option might be to exclude extinction from behavioral intervention packages aimed at decreasing interfering behavior, and a growing amount of research has evaluated this. For example, Ingvarsson et al. (2009) evaluated functionally arbitrary noncontingent reinforcement in the form of preferred tangibles with and without extinction for escape-maintained interfering behavior and found it was effective for two of three participants. However, stronger results were obtained with the addition of escape-extinction. Other research has produced mixed results, with some studies showing that extinction may be required in order to substantively thin out reinforcement for alternative behavior to clinically reasonable levels (Piazza et al., 1997), and other studies showing that treatment effects can be produced and maintained even in the absence of extinction, so long as the magnitude and quality of positive reinforcers are manipulated sufficiently (Briggs et al., 2019).

A recent systematic review of research on reducing escape-maintained behavior without escape extinction identified 38 peer-reviewed articles including 79 participants that omitted escape extinction (Chazin et al., 2021). Overall, across the studies, procedures that omitted escape extinction produced lower rates of target behavior than baseline phases and comparisons between procedures that included versus omitted escape extinction produced equivocal results. The authors interpreted these findings as supporting the potential utility of behavioral intervention procedures that do not include escape extinction. Although the results of this review are highly encouraging and intervention without extinction is certainly possible under some circumstances, it seems plausible that extinction may not be able to be eliminated entirely for some learners and some behaviors.

One alternative to omitting extinction would be to modify the way in which it is implemented to increase its acceptability. Piazza et al. (1996) evaluated differential positive reinforcement for compliance with escape extinction, with and without physical guidance, in an 11-year-old boy with autism. During escape extinction without physical guidance, demands were presented vocally and gesturally and physical guidance was not used. If the participant did not comply with instructional requests, researchers continued to present vocal and gestural requests continuously until compliance occurred, at which point the participant accessed positive reinforcement. If the participant left his seat, instructional demands were not continued, but he was reminded vocally every 2 min that he could earn positive reinforcement if he returned to his seat and completed his work. The number of instructional trials required before positive reinforcement was earned was gradually increased from 1 to 28. The procedure was highly effective and was compared to a subsequent escape extinction procedure that included physical guidance; escape extinction without physical guidance was more effective. Although this study with a single participant was promising, little additional research has attempted to replicate it and little research has been published that has attempted to modify extinction to make it more socially valid.

In popular psychology media, such as blogs or websites created for parents and other caregivers, caregivers are often given advice to respond to their child’s tantrums by validating the child’s feelings, which consists of delivering attention in the form of statements of concern (Bernstein, 2013). On one hand, responding to an upset child by trying to comfort them is likely a compassionate approach, and yet some behavior analysts may be concerned that responding to a tantrum with a preferred consequence, such as attention, may inadvertently reinforce the behavior. However, if the maintaining function of the behavior is not attention, then it may still be possible to implement functional extinction, while also providing a warm, empathetic, emotionally validating response to the child’s emotions and experience. If such attempts at comfort and emotional validation have the effect of alleviating suffering or distress for the child, then this procedure might be considered a more compassionate approach to extinction, in line with the definition of compassion offered by Taylor et al. (2019). To date, no research of which we are aware has attempted to evaluate the effectiveness of such a procedure. The connect and shape model of behavior management describes procedures similar to these (Whittingham, 2015), but no known research has been published that has evaluated the efficacy of that model.

The purpose of the current program evaluation was to empirically evaluate the effectiveness of real-life procedural modifications to extinction procedures that were being implemented at a community-based ABA provider. The purpose of the procedural modifications was to make extinction more “kind” by offering individualized authentic comfort and positive regard to the learner, while still functionally implementing extinction. The specific procedural modification evaluated in this study consisted of providing functionally arbitrary reinforcers in the form of positive attention, contingent on interfering behavior, while still implementing extinction for the identified function of the behavior (i.e., escape and tangible). In addition, we assessed the acceptability of the procedure to caregivers and staff.

## Method

### Participants and Setting

Four children with autism spectrum disorder, ages 3–4 years, participated in the study. All were receiving comprehensive ABA treatment from a community-based service provider, with a weekly intensity of 22, 27, 26, and 17 hr of one-on-one treatment for Agnes, Gilbert, Mildred, and Ingrid, respectively. All were referred for participation in this program evaluation because they engaged in behaviors that their clinical team identified as interfering with learning. All program evaluation sessions were conducted by the participants’ regular clinical team, in the context of their regular behavioral intervention services, which consisted of center-based services for Agnes, Gilbert, and Ingrid, and home-based services for Mildred.

### Response Measurement

Agnes engaged in a variety of behaviors that made it challenging for her to benefit from instruction, including vocal protests and tantrums (i.e., yelling or screaming in a voice volume above conversational level, crying, falling to the floor), tensing body, and throwing objects. Gilbert engaged in tantrums that interfered with learning, including screaming, crying, and falling to the ground. Ingrid and Mildred engaged in tantrums and vocal protesting that made it difficult for them to benefit from instruction.

Data were also collected on replacement behaviors for all participants. Replacement behaviors for all participants included mands for escape or tangible items. During all sessions, frequency data were collected on tantrums and manding using pen-and-paper data collection. Data were also collected on learner attempts to respond to clinician-initiated instruction. Learner responding to instructional trials was defined as the learner responding to a clinician instruction in a contextually appropriate manner within 5 s of the instruction, excluding mands or interfering behavior (which were measured separately) or other noncontextually related behaviors (e.g., repetitive behavior). Responses did not need to be correct to be scored as responses to instruction. For example, if a clinician asked a learner to name an object and they emitted the incorrect name, that response would still be scored as a response to the clinician’s instruction. Learner responding to instructional trials was scored as present or absent for each instructional trial and summarized as the percentage of instructional trials in which the learner responded to the instruction.

Social validity was measured by asking the caregivers of participants and the direct care staff working with the participants questions with Likert-type scales post-treatment. Table 1 depicts the questions and scores for each participant.

**Table 1:** Social validity data

	Respon-dent 1	Respon-dent 2	Respon-dent 3
Caregivers			
Changes made to my child’s extinction intervention made the intervention more kind	4	5	5
I believe the extinction intervention modeled by the team was effective in decreasing interfering behaviors	4	4	5
I prefer the modified extinction that was modeled over traditional extinction procedures	5	5	4
I would like to implement the modified extinction intervention with my child	4	4	3
I believe that giving comfort as a consequence for interfering behavior made the behavior worse for my child	2	1	1
I value the goal of making ABA procedures feel more kind to my child	5	5	5
Staff			
I approve of the goal of the modifications we implemented to make extinction procedures more kind	5	4	5
I believe the extinction procedures we used were effective in decreasing the clients’ interfering behaviors	5	4	5
I prefer the modified extinction procedures we implemented over traditional extinction procedures	5	4	5
I would like to implement the modified extinction procedures we used with other clients in the future	5	4	5
I believe that giving attention as a consequence for interfering behavior made the behavior worse for my client	1	2	3

Questions and scores for caregivers and staff involved in the study. Questions were answered from 1 (strongly disagree) to 5 (strongly agree)

### Interobserver Agreement

A second independent observer collected data to assess interobserver agreement (IOA). The total method was used to calculate IOA, by dividing the smaller frequency recorded by the larger frequency recorded for each session, dividing the smaller frequency by the larger frequency, and multiplying by 100. Table 2 depicts the percentage of sessions with IOA data, the mean IOA, and the range of IOA, for all measures and all participants.

**Table 2:** Interobserver Agreement (IOA) data

	% of Sessions	Mean IOA	Range of IOA
Agnes			
Tantrums	27	100	100–100
Mands	32	98	86–100
Response to instructions	29	100	100–100
Ingrid			
Tantrums	29	98	86–100
Mands	17	95	90–100
Response to instructions	177	100	100–100
Gilbert			
Tantrums	31	97	83–100
Mands	28	96	83–100
Response to instructions	29	100	100–100
Mildred			
Tantrums	32	100	100
Mands	32	94	88–100
Response to instructions	32	97	80–100

Percentage of sessions with IOA data, mean IOA, and range of IOA, for all measures and all participants

### Procedures

All participants received functional assessments, followed by evaluations of their kind extinction treatment programs.

#### Indirect Functional Assessment

An indirect functional assessment was conducted for Agnes’s behavior by using the Questions About Behavioral Function (Paclawskyj et al., 2000).

#### Functional Analysis

Interview-informed synthesized contingency analyses (IISCA), currently referred to as “practical functional assessments,” were conducted for Gilbert, Ingrid, and Mildred. IISCA procedures were similar to those described in Hanley et al. (2014).

#### Kind Extinction Treatment Evaluation

During all sessions in the treatment evaluation, participants were engaged with their regular learning activities, consisting of structured or naturalistic discrete trial instruction, depending on the individual participant’s regular treatment format. Sessions were 10 min in duration for Agnes and 5 min in duration for all other participants. Before each session began, a brief multiple stimulus preference assessment was conducted, consisting of a single choice trial among two or three highly preferred stimuli that had been identified as tangible reinforcers functionally related to the target interfering behavior. The following contingencies were in place for replacement behaviors during all baseline, kind extinction, and follow-up sessions: Learner responding to instructional trials and mands for the functional reinforcers, in the absence of target interfering behaviors, resulted in immediate access to the functional reinforcers for 30 s. Compliance with instructions was not physically prompted. Individual prompting and prompt fading hierarchies were determined by each participant’s clinical case supervisor, but generally included most-to-least prompting for skills that were on acquisition and least-to-most prompting for skills that were being maintained.

##### Baseline

Baseline sessions were identical to the test sessions of the IISCA, with the exception that replacement behaviors resulted in access to functional reinforcers, as described above.

##### Kind Extinction

During kind extinction sessions, replacement behaviors continued to be reinforced with the same synthesized contingency of escape-to-tangible on a fixed ratio 1 schedule, identical to baseline. Kind extinction procedures consisted of two components: (1) breaking the contingency between the target behaviors and access to escape and tangibles; and (2) providing immediate positive attention and validation (not the functional reinforcer) to the participant, contingent on the target behavior and any accompanying emotional responding. For example, contingent on tantrum behavior, the clinician may have offered their hand to hold and stated, “Cleaning up can feel really hard. I can see you’re frustrated.” The specific topographies of comfort and validation were customized for each individual participant, based on caregiver report and on direct observations by the clinical team of what acts of kindness resulted in positive affect (e.g., smiling, laughing) and movement toward clinicians in the past. In addition, during all sessions, if clinicians observed that an act of kindness resulted in a worsening of affect (e.g., grimacing, frowning) or moving away from the clinician, then the clinician chose a different act of kindness for the rest of the session. In addition, acts of kindness were offered, not forced. For example, hugs were offered gently, not physically forced.

To break the contingency between the target behavior and escape from work, clinicians implemented another instruction after delivering attention contingent on the target behavior. The same prompting hierarchies were implemented as were done in baseline (e.g., partial or full verbal models, gesture prompts, modeling prompts) again omitting physical prompting. If the learner eloped from the instructional area, the instructional materials were brought to the learner. To break the contingency between the target behavior and tangible reinforcers, after the clinician delivered validation contingent on the target behavior, they withheld the tangible reinforcer until the learner engaged in a replacement behavior in the absence of the target behavior.

##### Follow-up

After the kind extinction phase, the learner’s regular ongoing treatment team were trained in kind extinction and the treatment plan was then implemented across the remainder of the learner’s regular behavioral technicians. Formal evaluation data were discontinued until follow-up data were collected to evaluate if treatment gains had maintained after 2 weeks for Agnes, 3 weeks for Ingrid, 4 weeks for Gilbert, and 4 weeks for Mildred. Contingencies during follow-up sessions were identical to the kind extinction phase.

## Results

**Fig. 1:**
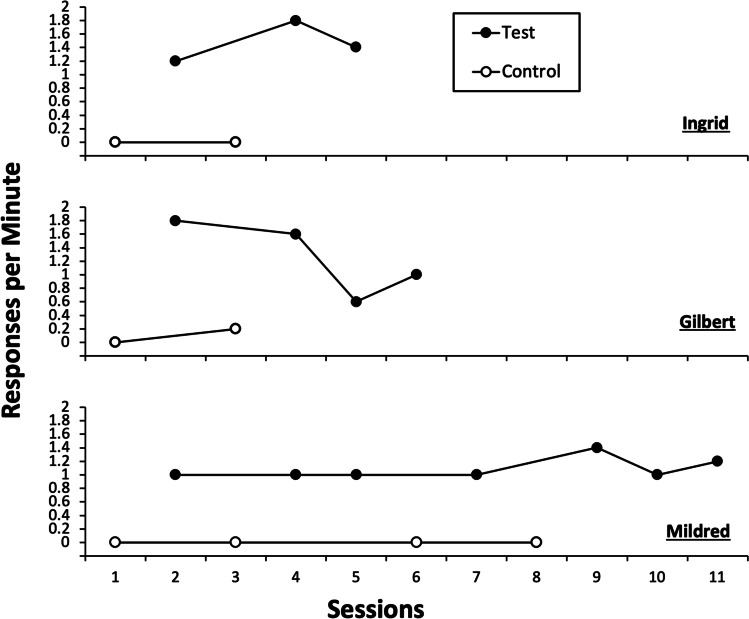
Practical Functional Assessment (PFA) Results. *Note.* Black circles depict rate of tantrums and precursor behavior in the synthesized tangible and escape condition, whereas white circles depict rate of tantrums and precursor behavior during the control condition

The results of the indirect functional assessment for Agnes suggested escape and tangible functions for her behavior (data available from authors upon request). Figure 1 depicts the results of the IISCA for Mildred, Ingrid, and Gilbert. The results of the IISCA suggested a synthesized function of escape-to-tangible for Mildred, Ingrid, and Gilbert.

**Fig. 2:**
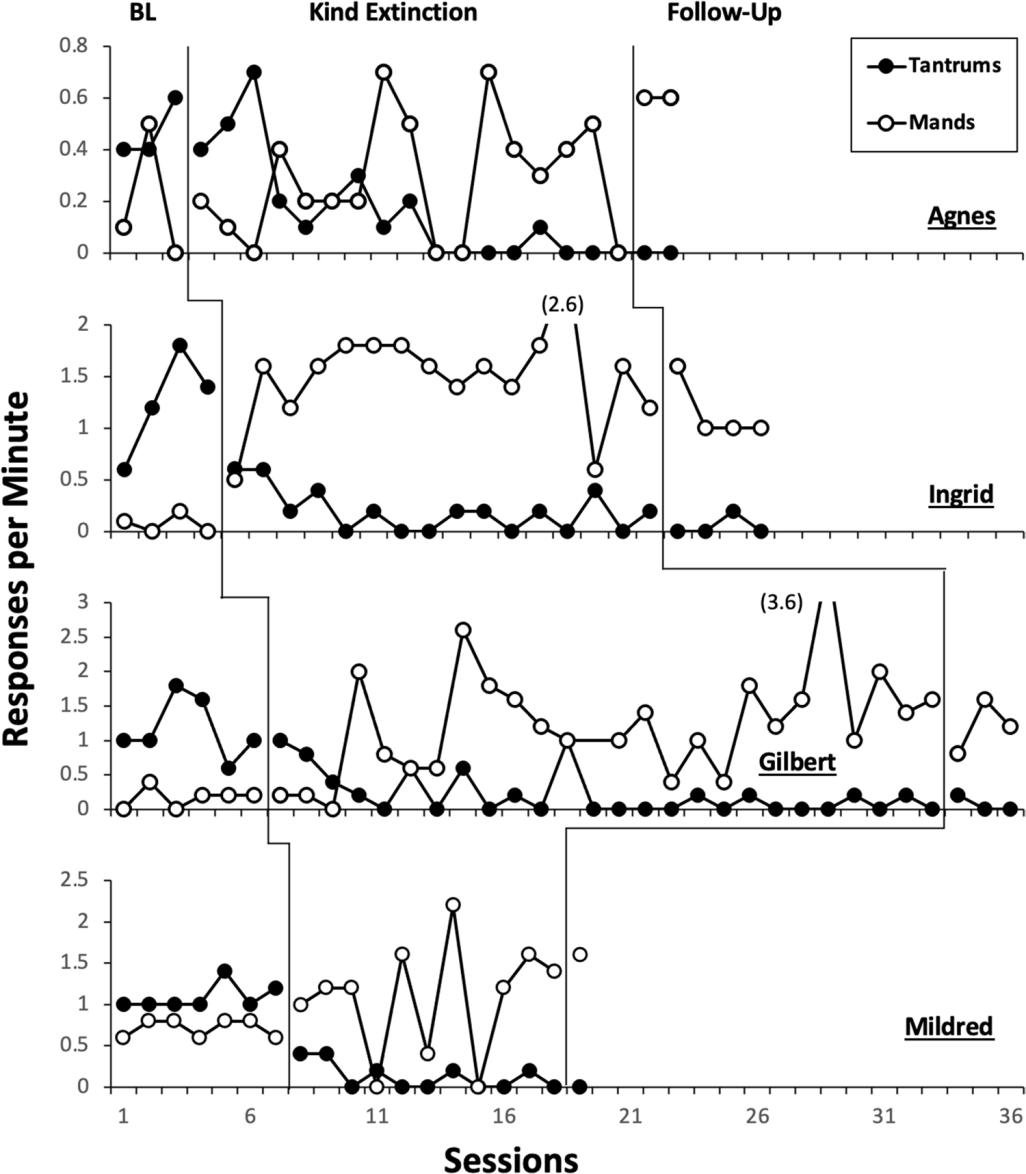
Kind Extinction Treatment Evaluation. *Note.* Black circles depict rate of tantrums and white circles depict rate of mands during baseline, kind extinction, and follow-up conditions, across all participants

Figure 2 depicts responses per minute of tantrums and mands during the kind extinction treatment evaluation for all participants. During the baseline phase, increasing or stable rates of tantrums were observed for all participants, with a mean of .47, 1.25, 1.17, and 1.09 response per minute for Agnes, Ingrid, Gilbert, and Mildred, respectively. Substantial decreases in tantrums were observed during the kind extinction phase for all participants, with a mean of .18, .2, .22, and .13 response per minute for Agnes, Ingrid, Gilbert, and Mildred, respectively. The mean percentage decrease compared to baseline in the kind extinction condition was 62%, 84%, 81%, and 88% for Agnes, Ingrid, Gilbert, and Mildred, respectively.

During follow-up, mean rates of tantrums were 0, .05, .07, and 0 responses per minute for Agnes, Ingrid, Gilbert, and Mildred, respectively. Follow-up rates represent a mean reduction from baseline of 100%, 96%, 94%, and 100% for Agnes, Ingrid, Gilbert, and Mildred, respectively.

Manding was generally low and/or variable during baseline for all participants, with mean rates of .2, .08, .14, and .7 responses per minute for Agnes, Ingrid, Gilbert, and Mildred, respectively. During the kind extinction phase, their average mands per minute increased to .3, 1.5, 1.24, and 1.07, respectively. During follow-up, mands occurred at a mean rate of .4, 1.15, 1.2, and 1.6, respectively.

**Fig. 3:**
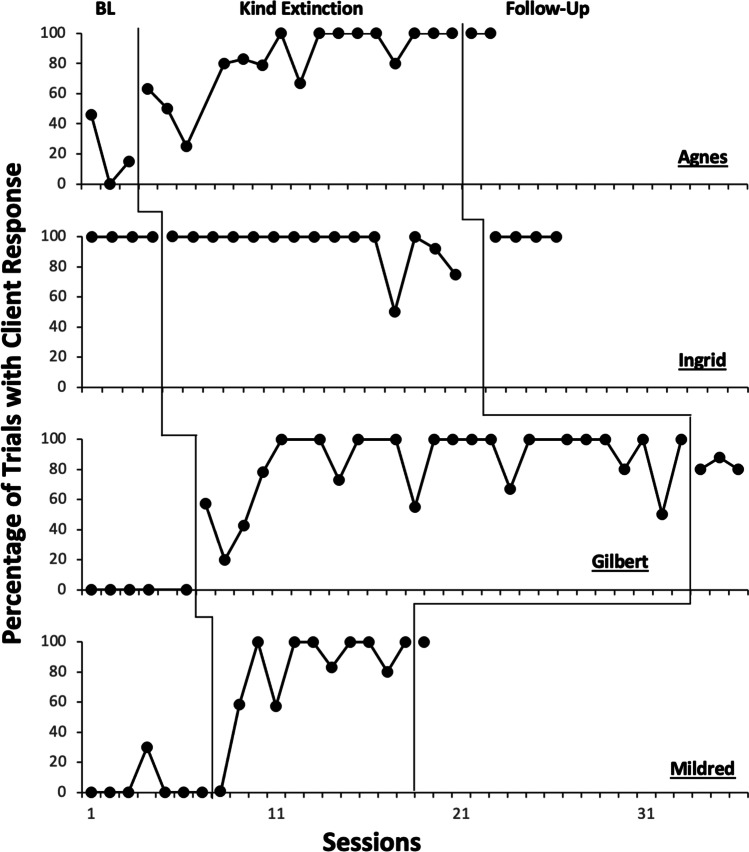
Participant Engagement with Instruction. *Note.* Data depict the percentage of clinician-delivered instructional trials in which participants responded to instruction during baseline, kind extinction, and follow-up conditions, across all participants

Figure 3 depicts the percentage of trials in which all participants responded to clinician-initiated teaching trials for all participants. Agnes, Gilbert, and Mildred displayed low percentages of response-to-instruction during baseline, with mean percentages of 20%, 0%, and 4%, respectively. All three demonstrated substantial increases in the behavior during the kind extinction phase, with means of 84%, 84%, and 88%, respectively. During follow-up, they responded to clinician-initiated instructional trials during 100%, 83%, and 100% of opportunities. Ingrid responded to clinician-initiated teaching trials during 100% of trials in the baseline phase and her responding remained high throughout the rest of the evaluation, with 94% during kind extinction and 100% during follow-up.

## Discussion

Rapid and substantial decreases in interfering behavior were observed across all four children who participated. Although not directly targeted in the intervention, replacement behaviors in the form of mands and responding to instruction also substantially improved. Furthermore, follow-up data were collected and the effects of treatment maintained at 2–4 weeks. These results provide preliminary evidence suggesting that providing genuine positive attention and emotional validation while implementing escape and/or tangible extinction may be an effective procedure for decreasing interfering behavior in some children with autism.

In addition to being effective, the data on social validity provide initial evidence that the kind extinction procedure was found acceptable to both caregivers and staff. In an open-ended written comment to their child’s clinical staff, one caregiver wrote that “I think it helps the kids feel loved and if we just ignore them they may feel unloved.” It is interesting to note that one staff member, respondent 3 in Table 1, provided a score of 3 (neither agree nor disagree) to the question of whether providing verbal attention during kind extinction made the behavior worse for their child. However, they also followed up with open-ended typed feedback that stated the following, “Vocal attention didn’t work but rubbing his back seemed to calm him down during a task. Overall, I found modified extinction to be successful.”

In response to the question asking parents and staff whether they preferred kind extinction over traditional extinction, all participants responded positively, with either a score of 4 or 5. These social validity data provide preliminary evidence that kind extinction may be more socially acceptable than traditional extinction. This finding could be important because people may be more likely to adhere to treatments that they find more acceptable (Milosevic et al., 2015). However, these data should be interpreted with caution because the current study only implemented kind extinction. Parents had previous experience with traditional extinction before participating in this program evaluation but their experience with kind extinction was more recent at the time that they answered the social validity questions. Staff had previous experience with traditional extinction and were concurrently implementing traditional extinction with other learners who did not participate in this program evaluation. Future research could compare the social validity of traditional versus kind extinction in people who have an equal duration of experience with each.

In addition to the effectiveness and social validity of kind extinction, BCBAs are ethically bound to consider how intrusive and how compassionate our procedures are (Behavior Analyst Certification Board, 2020). We might elaborate on the core principle of treating others with compassion by suggesting that creating nurturance and support for our learners, even during the most difficult of times, is the purpose of what we do in ABA. Behavioral principles and procedures, in themselves, are capable of being implemented more-ethically or less-ethically, as well as more-compassionately and less-compassionately. It may be reasonable to argue that the core principle of treating others with compassion calls on us to consider how compassionate a procedure is as equally important to how effective it is and how socially valid it is. Much more future research is needed to innovate and refine ABA procedures to make a variety of our procedures more compassionate.

It should be noted that compassion, as defined by Taylor et al. (2019), consists of actions that alleviate the suffering or distress of others. We did not directly evaluate whether the extinction procedures used in this program evaluation decreased distress for the children who participated. Future research might include measures of affect so that clinicians may quantify whether implementing kind extinction increases, decreases, or does not change client distress (Reid, 2016). Such data might help evaluate the extent to which offering emotional validation and kindness during extinction may have the effect of being a more compassionate approach to extinction.

One potential concern with providing attention contingent on interfering behavior is that we may reinforce the behavior with attention, especially if the behavior is already partially maintained by attention. If the behavior is not already partially maintained by attention, then one concern is that we may inadvertently “shape up” an attention function. In either case, if providing attention contingent on behavior while implementing tangible and/or escape extinction did indeed reinforce the behavior then, by definition, the behavior would either persist or increase in rate. This was not observed in any of the four participants in the current study. An additional concern could be that contingent attention could somehow encourage attention-maintained interfering behavior outside of study sessions but this concern does not seem plausible. If a particular consequence is reinforcing a behavior then the effect on that behavior is far more likely to be seen in the context in which the consequence is delivered, as compared to some other unrelated context in which that reinforcer is not delivered. In short, if contingent attention did indeed reinforce the target behaviors in this study, the most logical place that would have been observed was during the treatment sessions that the data reflect.

If future practitioners are concerned about potentially “shaping up” an attention function, then implementing this procedure and measuring the effects, as we did in the current program evaluation, would give the practitioner the tools they would need to detect that effect and discontinue the procedure. Put simply, if a practitioner tried the kind extinction procedure and the behavior did not decrease, either because it did not functionally represent extinction or because it “shaped up” an attention function, then that would be clearly visible in the treatment evaluation data: The rate of behavior would not decrease. The procedure could then be terminated in favor of a more traditional extinction procedure or another alternative.

When motivating operations are considered, it is perhaps not surprising that providing contingent attention while implementing kind extinction did not reinforce the behavior. By definition, when tangible and/or escape-motivated interfering behavior occurs, a tangible and/or escape establishing operation must be in place for those reinforcers to be potent at that time (Laraway et al., 2003). Therefore, it seems probable that a powerful establishing operation for attention was lacking, which should predict the lack of a positively reinforcing effect of attention on interfering behavior in that context.

Some limitations to the current study warrant discussion. First, we did not conduct an experimental functional analysis for one of the four participants and instead used only indirect assessment methods. Future research should consider including experimental functional assessment methods for all participants. One related potential concern is that because we conducted synthesized functional analyses for three participants, and did not rule out attention specifically, it is possible that the interfering behavior of some or all of the participants had attention functions. This possibility seems particularly unlikely because, if the behaviors were sensitive to reinforcement by attention, then the treatment procedure should have maintained the behavior, as attention was always given contingent on interfering behavior.

An additional potential limitation is that we did not compare kind extinction to more traditional extinction procedures, so it is possible that more traditional procedures would have produced larger or more rapid reductions in interfering behavior. Although this is possible, we believe that the reductions observed in the current study were amply rapid and large to be clinically significant. In addition, the social validity data suggest that the procedure was highly acceptable and, one might argue, that kinder approaches to extinction do not need to out-perform traditional extinction, as long as they are effective and stakeholders find them socially valid. Still, future research might attempt to evaluate whether kind approaches to extinction may actually outperform traditional approaches, such as leading to lesser extinction bursts.

Finally, one potential limitation is that we implemented kind extinction on top of a baseline of differential reinforcement; that is, we did not test kind extinction alone. From a scientific perspective, it would be desirable to evaluate a procedural modification of extinction alone. From a practical perspective, such an evaluation may be of little utility, because extinction is almost never considered an adequate behavior intervention plan in isolation anymore (Cooper et al., 2019). The specific reason we chose to not evaluate kind extinction in isolation is that the evaluation was done in the context of real-life everyday treatment for learners in our care and we had no ethically justifiable reason to implement extinction alone. Still, because we only evaluated kind extinction in combination with differential reinforcement of alternative behavior, future research could consider evaluating kind extinction procedures in isolation, if ethical, or in combination with other ethical intervention components.

One detail the clinicians in this treatment evaluation anecdotally noted was that they appreciated how the kind extinction procedure gave them something to actively do while implementing extinction, in addition to not reinforcing the behavior with the functional reinforcer. In other words, it seems possible that the kind extinction procedure may function as a type of alternative behavior for staff or caregivers to engage in, instead of merely omitting reinforcement for the target behavior. Future researchers may consider evaluating whether giving caregivers an alternative behavior, such as providing positive attention, increases the integrity with which caregivers withhold the functional reinforcer when being trained to implement extinction with their children. It also seems possible that providing positive attention may provide staff or caregivers with a behavior that allows them to escape from implementing the potentially nonpreferred procedure of following through with instructional requests. However, if the kind extinction procedure is less aversive for staff or parents to implement, then implementing it should create less of an establishing operation that evokes staff avoidance of implementing the procedure. In other words, higher-preferred extinction procedures may be less motivating for staff to avoid and therefore could lead to higher procedural integrity on the part of staff or caregivers. This possibility remains purely speculative and should be addressed by future research.

We do not suggest that providing positive regard as a consequence of interfering behavior is the only or best way to implement extinction more kindly. Perhaps having a discussion with the learner before implementing extinction, getting input from them on the form or format of extinction they would prefer, or empowering the learner to choose or control some aspect of the extinction procedure could also make extinction more kind, while maintaining equal or better effectiveness. In particular, future research could consider evaluating the social validity of kind extinction from the learner’s perspective. Treatment choice procedures (e.g., Hanley, 2010; Rajaraman et al., 2022) could be used to assess whether learners, even ones who do not yet have a functional manding repertoire, consistently choose kind extinction over traditional extinction.

Future research is clearly needed on procedures for implementing attention extinction with more kindness. At the most fundamental level, attention extinction can easily be labeled as “ignoring” a person (even if it is rationalized by specifying that the behavior, not the person, is being placed on extinction) and it is plainly apparent that ignoring another human being can appear rude, cold, and uncaring. Future research might evaluate an attention extinction procedure where, contingent on the target behavior, the learner is briefly and kindly told something like, “I’m really sorry, I know you need my attention right now but I can’t give it to you,” followed by then providing no attention until the interfering behavior ceases for a specified unit of time. Such a procedure may not actually be extinction at all, but rather, a small amount of attention reinforcement contingent on the target behavior. Some previous research has manipulated the parameters of attention reinforcement for target behavior versus alternative behaviors to make the attention reinforcement for alternative behaviors more potent than the attention reinforcement for the target behavior. For example, Athens and Vollmer (2010) implemented higher preferred attention (praise) contingent on alternative behavior and lower-preferred attention (reprimands) contingent on the target behavior and successfully produced a reduction in the target behavior.

In conclusion, if and when extinction is necessary, the manner in which it is commonly implemented may appear cold and uncaring to some. Especially for caregivers of children with interfering behavior, traditional extinction may be perceived and described as “The behavior analyst won’t let me comfort my child when they are upset.” If such procedures are indeed *the only way* to decrease behavior in a socially meaningful manner, and research has been published to substantiate that, then it may indeed be worth it to ask caregivers to make this sacrifice. But if it is possible to do what we do with greater kindness and compassion, one might consider it an ethical imperative to at least try.

As the field of ABA takes stock of our daily practices and considers them from a perspective of compassion and kindness, we might ask ourselves questions such as, Would I want a professional ignoring my child when they are genuinely upset? Would I want someone to ignore *me* when I am genuinely upset, even if I am behaving maladaptively? Can I remember a time in my own life when I was really struggling and genuinely felt scared or unsupported by others? Are we okay with potentially allowing our learners and their caregivers to feel that way? As the field of ABA grapples with decades-long challenges with public perceptions of our field, it may be more important than ever to consider ways in which we can be equally effective at what we do, but more humane in how we do it. The fundamental question that spurred the treatment evaluation project that this article describes was: Can we be more kind while also holding fast to the foundational dimensions that define our science and make our practical work so powerful? We fundamentally believe that the answer to this question is a resounding yes and, at the same time, much work is left to be done in this direction. The current article describes just one small step toward reevaluating foundational ABA procedures with an aim toward increasing compassion and kindness in everything we do. We hope that this small step will encourage other researchers and practitioners to continue this journey with creativity and humility.

## Data Availability

The datasets generated during and analyzed during the current study are available from the corresponding author on reasonable request.
